# Protruding Aortic Arch Thrombus in Cancer-Associated Stroke: Caution Regarding Endovascular Access

**DOI:** 10.1016/j.jaccas.2025.105489

**Published:** 2025-09-17

**Authors:** Yasufumi Gon, Tomohiro Kawano, Junji Takasugi, Hideaki Kanki, Tsutomu Sasaki, Hideki Mochizuki

**Affiliations:** a Department of Neurology, Graduate School of Medicine, The University of Osaka, Osaka, Japan; b Department of Medical Innovation, Academic Clinical Research Center, The University of Osaka Hospital, Osaka, Japan; c StemRIM Institute of Regeneration-Inducing Medicine, Graduate School of Medicine, The University of Osaka, Osaka, Japan

**Keywords:** cancer, endovascular therapy, stroke, thrombus

## Abstract

**Background:**

In cancer-associated stroke, thrombi can be present beyond the cerebral vasculature. We report a case in which a protruding thrombus in the aortic arch was identified during the thrombectomy time window.

**Case Summary:**

A 65-year-old woman with stage IIIC2 endometrial cancer presented with aphasia and right hemiparesis. Magnetic resonance imaging showed acute cerebral infarction in the left temporal lobe with M2 occlusion. Contrast-enhanced imaging revealed multiple thrombi, including a protruding thrombus in the aortic arch as well as others in the celiac artery, pulmonary artery, and femoral veins. Although the patient was eligible for endovascular therapy, the procedure was deferred owing to high risk of further embolization.

**Discussion:**

Endovascular therapy is a potential option in cancer-associated stroke but requires caution when aortic arch thrombi are present owing to increased embolic risk.

**Take-Home Message:**

Comprehensive vascular imaging is essential in cancer-associated stroke to guide treatment and avoid procedural complications.

## Graphical Abstract

**Figure:**
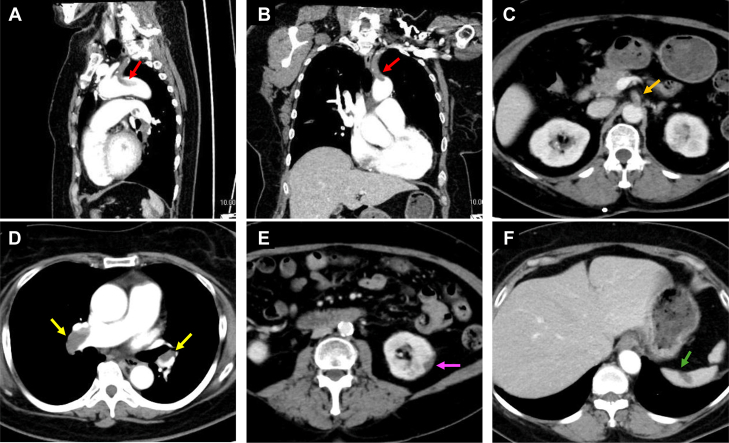

## History of Presentation

A 65-year-old woman was diagnosed with uterine cancer 5 months before presentation. Four months before presentation, she was referred to our gynecology department and underwent total laparoscopic hysterectomy with bilateral salpingo-oophorectomy and lymph node dissection. Surgical findings classified the endometrial cancer as T1bN2M0, stage IIIC2. She was prescribed adjuvant paclitaxel/epirubicin/carboplatin therapy.

**Take-Home Message**

• Comprehensive vascular assessment is crucial in cancer-associated stroke to identify thrombi along the catheter access route and guide treatment decisions, as conservative anticoagulation may be preferable to endovascular therapy when high embolic risk is present.

**Figure 1:**
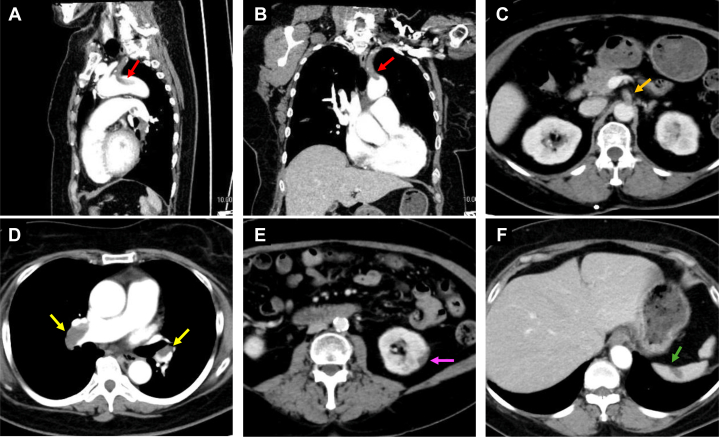
Contrast-Enhanced Computed Tomography Findings at the Time of Stroke Center Consultation (A and B) Thrombi were observed extending from the left subclavian artery to the aortic arch, with protruding thrombi in the aortic arch (red arrows). (C to F) Multiple thrombi were present in the celiac artery (C, orange arrow) and pulmonary arteries (D, yellow arrow), with evidence of renal infarction (E, purple arrow) and splenic infarction (F, green arrow).

The patient was admitted for a scheduled third cycle of chemotherapy. Upon admission, contrast-enhanced computed tomography was performed to evaluate the tumor and revealed multiple thrombi, including in the aortic arch (Figures 1A and 1B), celiac artery (Figure 1C), pulmonary artery (Figure 1D), and renal (Figure 1E) and splenic infarctions (Figure 1F). Thrombi were also observed in both femoral veins. Blood tests showed markedly elevated D-dimer levels (8.1 μg/mL). At this time, the patient exhibited no subjective symptoms of thromboembolism. She was diagnosed with cancer-associated thrombosis (CAT), and continuous infusion of unfractionated heparin (10,000 U/d) and aspirin (100 mg/d) were initiated under the attending physician's guidance.

On hospital day 7, her condition was stable until 21:00, but by 23:30, she had exhibited reduced responsiveness to verbal stimuli, prompting a stroke team consultation.

## Past Medical History

The patient's past medical history included hypertension and dyslipidemia, both managed with antihypertensive medication and statins. She reported no history of alcohol consumption or smoking. She had completed 2 cycles of chemotherapy without complications.

## Investigations

**Figure 2:**
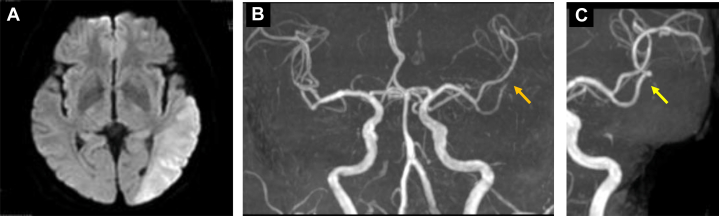
Magnetic Resonance Imaging Findings at the Time of Stroke Center Consultation (A) Magnetic resonance imaging revealed acute cerebral infarction in the left temporal lobe, and (B) magnetic resonance angiography demonstrated occlusion of the left middle cerebral artery M2 segment (orange arrow). (C) Follow-up imaging after 1 week of anticoagulation therapy showed recanalization of the occluded vessel (yellow arrow) without new brain infarction.

Examination revealed a Japan Coma Scale score of I-3, blood pressure of 180/100 mm Hg, oxygen saturation of 99% on room air, and body temperature of 36.5 °C. Electrocardiogram showed no arrhythmias. Neurological examination identified fluent aphasia and right-sided weakness, with a National Institutes of Health Stroke Scale score of 8. Magnetic resonance imaging confirmed acute ischemic stroke (AIS) in the left parietal cortex (Figure 2A), and magnetic resonance angiography showed M2 segment occlusion of the left middle cerebral artery (MCA) (Figure 2B).

## Management

Although within the endovascular treatment (EVT) time window, the procedure was deemed high risk because of multiple thrombi along the access route as seen on contrast-enhanced computed tomography, and conservative management was continued. After 1 week of antithrombotic therapy, follow-up imaging showed thrombus resolution (Figure 2C). Anticoagulation was transitioned to apixaban, and the patient was transferred to a rehabilitation facility.

## Outcome and Follow-Up

Three months after onset, the patient's functional outcome improved to a modified Rankin scale score of 2, with no recurrent stroke or major bleeding. Five years after the ischemic stroke, she remains on apixaban therapy without recurrence. Additionally, her uterine cancer treatment continues to progress favorably.

## Discussion

This case highlights the unique challenges of managing AIS in cancer patients. Ischemic stroke represents the most common arterial thromboembolic complication in this population,^1^^,^^2^ and although recent studies show that patients with cancer achieve similar EVT outcomes compared with those without cancer,^3^^,^^4^ they may develop thrombi in multiple arterial territories beyond the intracranial vessels. When thrombi are present along the catheter access route, the endovascular procedure could lead to unexpected multiple embolization. Although the patient was within the time window of EVT, the procedure was deferred owing to high-risk protruding thrombi in the aortic arch. Conservative treatment followed by transition to oral anticoagulation resulted in a favorable outcome. Our case underscores the importance of thoroughly inspecting the catheter access route for thrombi in cancer patients before thrombectomy.

Advances in reperfusion therapy have improved AIS outcomes, and EVT within 6 hours is now the standard treatment for occlusions of the internal carotid or M1 MCA segments.^5^^,^^6^ A recent meta-analysis indicates that EVT may benefit proximal M2 segment occlusions, suggesting a potential expansion of EVT indications.^7^ However, in patients with concurrent cancer, careful risk assessment is essential. As demonstrated in our case, the presence of protruding thrombi along the catheter access route poses a high risk of thromboembolism in other territories, potentially leading to severe complications. Therefore, thorough assessment of the entire vascular system, especially the access route, is essential before proceeding with EVT in patients with AIS and active cancer.

Performing EVT without evaluating the presence of thrombi in the aortic arch can result in recurrent ischemic stroke. Johno et al^8^ reported a case of recurrent stroke after thrombectomy for AIS with MCA occlusion, in which a floating thrombus was identified in the aorta. Although their case did not involve cancer, it demonstrates that aortic thrombi can cause complications during EVT. In patients with cancer, who are in a hypercoagulable state, as demonstrated in our case, this risk may be even higher.

Our patient was treated with heparin anticoagulation and transitioned to apixaban, with no stroke recurrence during follow-up. Unfractionated heparin was used because low-molecular-weight heparin is unavailable for CAT treatment in Japan. For secondary prevention of cryptogenic stroke in cancer patients, no significant differences have been observed in stroke recurrence or bleeding events between aspirin and apixaban.^9^ Furthermore, studies comparing edoxaban and enoxaparin have indicated no difference in D-dimer levels after stroke onset.^10^ Although further research is needed to identify the optimal anticoagulation therapy for cancer-associated stroke, direct oral anticoagulants may be a promising option.

Although this case demonstrates the importance of comprehensive vascular assessment in cancer-associated stroke, several clinical considerations merit discussion. First, in AIS, minimizing the time to thrombectomy is critical for favorable outcomes. Aortic evaluation is important, however it should not delay treatment. Routine inclusion of aortic assessment during contrast-enhanced computed tomography scans may help address this issue. Second, although our case had a favorable outcome with conservative management, evidence supporting such an approach in similar cases is lacking. EVT is the guideline-recommended treatment for AIS with large vessel occlusion within 6 hours of onset.^5^^,^^6^ Consequently, mechanical thrombectomy is performed in most eligible patients unless medical considerations suggest otherwise or unless declined by the patient or their family (eg, in end-stage disease or owing to economic or personal reasons). Although EVT remains the standard of care, our case suggests that individualized decision-making is warranted for cancer patients with extensive arterial thrombi, where procedural risks may outweigh the potential benefits. Further research and discussion are necessary to establish best practices.

## Conclusions

This case illustrates the complexity of treatment decisions in cancer-associated AIS when multiple thrombi are present throughout the vascular system. Although EVT has proven effective for cancer patients, with outcomes comparable to noncancer patients, the presence of protruding thrombi along the catheter access route necessitates careful risk-benefit assessment. Our patient's favorable outcome with conservative anticoagulation therapy demonstrates that deferring EVT may be appropriate in selected high-risk cases. Comprehensive preprocedural vascular imaging is essential to identify thrombi beyond the cerebral circulation and to guide treatment decisions. This approach may help prevent procedural complications while maintaining the potential for good functional outcomes through alternative therapeutic strategies.

## Funding Support and Author Disclosures

This work was supported by the 10.13039/501100001691Japan Society for the Promotion of Science (10.13039/501100001691JSPS) KAKENHI (grant number: JP23K09713). The authors have reported that they have no relationships relevant to the contents of this paper to disclose.
